# Prevalence of arthritis according to age, sex and socioeconomic status in six low and middle income countries: analysis of data from the World Health Organization study on global AGEing and adult health (SAGE) Wave 1

**DOI:** 10.1186/s12891-017-1624-z

**Published:** 2017-06-21

**Authors:** Sharon L. Brennan-Olsen, S. Cook, M. T. Leech, S. J. Bowe, P. Kowal, N. Naidoo, I. N. Ackerman, R. S. Page, S. M. Hosking, J. A. Pasco, M. Mohebbi

**Affiliations:** 1 Deakin University, Geelong, Australia; 2 Australian Institute for Musculoskeletal Science (AIMSS), The University of Melbourne-Western Precinct, Level 3, Western Centre for Health Research and Education (WCHRE) Building, C/- Sunshine Hospital, Furlong Road, St Albans, Melbourne, VIC 3021 Australia; 3 Department of Medicine-Western Health, Melbourne Medical School, The University of Melbourne, Melbourne, Australia; 4 Institute of Health and Ageing, Australian Catholic University, Melbourne, Australia; 5 Faculty of Medicine, Nursing and Health Sciences, Monash University, Melbourne, Australia; 6 Department of Health Statistics and Information Systems, World Health Organization, Geneva, Switzerland; 7 Research Centre for Generational Health and Ageing, University of Newcastle, Newcastle, Australia; 8 Department of Epidemiology and Preventive Medicine, Monash University, Melbourne, Australia; 9 Barwon Centre for Orthopaedic Research and Education, Barwon Health, Geelong, Australia

**Keywords:** Arthritis, Epidemiology, Prevalence, Socio-demographic characteristics, Low and middle income countries

## Abstract

**Background:**

In higher income countries, social disadvantage is associated with higher arthritis prevalence; however, less is known about arthritis prevalence or determinants in low to middle income countries (LMICs). We assessed arthritis prevalence by age and sex, and marital status and occupation, as two key parameters of socioeconomic position (SEP), using data from the World Health Organization Study on global AGEing and adult health (SAGE).

**Methods:**

SAGE Wave 1 (2007–10) includes nationally-representative samples of older adults (≥50 yrs), plus smaller samples of adults aged 18-49 yrs., from China, Ghana, India, Mexico, Russia and South Africa (*n* = 44,747). Arthritis was defined by self-reported healthcare professional diagnosis, and a symptom-based algorithm. Marital status and education were self-reported. Arthritis prevalence data were extracted for each country by 10-year age strata, sex and SEP. Country-specific survey weightings were applied and weighted prevalences calculated.

**Results:**

Self-reported (lifetime) diagnosed arthritis was reported by 5003 women and 2664 men (19.9% and 14.1%, respectively), whilst 1220 women and 594 men had current symptom-based arthritis (4.8% and 3.1%, respectively). For men, standardised arthritis rates were approximately two- to three-fold greater than for women. The highest rates were observed in Russia: 38% (95% CI 36%–39%) for men, and 17% (95% CI 14%–20%) for women. For both sexes and in all LMICs, arthritis was more prevalent among those with least education, and in separated/divorced/widowed women.

**Conclusions:**

High arthritis prevalence in LMICs is concerning and may worsen poverty by impacting the ability to work and fulfil community roles. These findings have implications for national efforts to prioritise arthritis prevention and management, and improve healthcare access in LMICs.

**Electronic supplementary material:**

The online version of this article (doi:10.1186/s12891-017-1624-z) contains supplementary material, which is available to authorized users.

## Background

Worldwide, musculoskeletal disorders represent a global threat to healthy ageing [1], and are ranked as the second most common cause of disability, measured by years lived with disability (YLDs) [2]. Lower and middle income countries (LMICs) are not immune to the burden of musculoskeletal diseases, indeed the prevalence of this non-communicable disease (NCD) group is dramatically increasing in LMICs [3]. The 2010 Global Burden of Disease (GBD) study reported that musculoskeletal diseases accounted for 19.2% of all YLDs in LMICs [3]. Despite this, the majority of the global NCD initiatives do not include musculoskeletal diseases [3]. Significantly contributing to the global disability burden associated with the musculoskeletal system are arthritis diseases. Arthritis is an umbrella term that encompasses in excess of 100 different arthritic conditions which are a chronic, painful, and debilitating group of diseases. Arthritis, specifically osteoarthritis, is a significant contributor to global disability burden, and the YLDs attributable to osteoarthritis have increased by 75% from 1990 to 2013 [2], indicating this disease as a growing problem internationally. In combination with an increasing trajectory of arthritis prevalence [2, 4], growth in YLDs attributable to arthritis is due primarily to increased life expectancy worldwide, and prolonged exposure to arthritis risk factors [5].

Compared to higher income countries, many LMICs [6], where two-thirds of the world’s population resides, have a much lower capacity to pay for adequate healthcare. Indeed, LMICs have 90% of the global burden of disease but only 12% of global health spending [7]. In higher income countries, arthritis is associated with reduced workplace productivity [8, 9]; however, for residents of LMICs, arthritis imposes a potential additional burden by creating a vicious cycle that subsequently worsens poverty [10]. For example, compared to higher income countries, and in context of scarce medical and social support systems, residents of LMICs with arthritis also experience reduced ability to access, afford or utilize treatments including analgesic and anti-inflammatory pharmacotherapies [11, 12], or arthroplasty for advanced disease [13, 14]. They also have, in context of workforce capacity limitations, less flexibility regarding working conditions or hours [15], and few if any options for early retirement, or social security ‘safety nets’ pertaining to minimum income, including financial and/or material goods.

Whilst the majority of research regarding arthritis prevalence has been undertaken in higher income countries, recent data from the 2010 GBD Study provides some evidence that LMICs may have greater arthritis prevalence than higher income countries [16]. Yet, while valuable population level estimates, extrapolation from these GBD estimates is difficult given that they are based on published prevalence and incidence data from a small number of heterogeneous studies spanning different time periods in a limited number of LMIC [17]. Furthermore, data from multi-country studies of LMICs that have examined prevalence of arthritis across sociodemographic factors are typically not readily available [18, 19], with the exception of a recent publication, which showed that more years of schooling and greater levels of wealth decreased the odds of having an undiagnosed NCD, including arthritis [20]. Understanding the prevalence of arthritis across different parameters of socioeconomic position (SEP) data would augment our global understanding of global arthritis prevalence, social determinants and burden.

To date, country-specific arthritis prevalence across parameters of SEP has not been systematically evaluated in large, nationally representative samples of populations from LMICs. This information is crucial for planning future healthcare delivery for high burden chronic conditions and to ensure sufficient health workforce capacity – both significant concerns in an ageing world [21]. Comprehensive data have been collected in the World Health Organization (WHO) Study on global AGEing and adult health (SAGE) [20, 22, 23], thus providing an important resource to investigate disease prevalence in large population samples from six LMICs. Using SAGE Wave 1, these analyses were undertaken to determine the prevalence of arthritis in LMICs according to age, sex, and socioeconomic position (SEP).

## Methods

### Study population and design

SAGE Wave 1 (2007–10) is a longitudinal study with nationally representative samples of persons aged 50+ years and a smaller sample of adults aged 18–49 years that includes 44,747 adults aged ≥18 years from China, Ghana, India, Mexico, Russian Federation and South Africa [23]. Multistage cluster sampling strategies were used with households as sampling units. Households were classified into one of two mutually exclusive categories: i) all persons aged 50 years and older were selected from “older” households, and ii) one person aged 18–49 years was selected from each “younger” household. An older or younger household was defined by the age of the respondent targeted for individual interview. Household-level and person-level analysis weights were calculated for each country. This research was performed in accordance with the Declaration of Helsinki. The WHO and the respective implementing agency in each country provided ethics approvals. Written, informed consent was obtained from all participants.

### Data collection in WHO SAGE

Using a standardized survey instrument to ensure consistency, and based on standardized methods, interviewer training and translation protocols, face-to-face interviews were conducted in China (2008–10; response 93%), Ghana (2008–09; response 81%), India (2007–08; response 68%), Mexico (2009–10; response rate 53%), the Russian Federation (2007–10; response 83%) and South Africa (2007–08; response 75%), as previously published [23]. Full details regarding the probability sampling design, cluster sampling strategies and country-specific areas included in SAGE have been published elsewhere [23]. Briefly, the SAGE questionnaire consisted of household, individual and proxy questionnaires, a verbal autopsy, and appendices: the domains of which are summarised in Table 1 [23].

**Table 1:** Questionnaire sections included in the SAGE Wave 1 standardized survey instrument [23]

Questionnaire section	
Household roster	Questions regarding the dwelling, income, transfers [of family members] in and out of the household, assets and expenditures
Individual questionnaire	Questions regarding health and its determinants, disability, work history, risk factors, chronic conditions, caregiving, subjective well-being, health care utilization and health systems responsiveness
Proxy questionnaire	Questions regarding health, functioning, chronic conditions, and health care utilization
Verbal autopsy	Performed to ascertain the probable cause of death for deaths in the household in the 24 months prior to interview or between interview waves
Appendices	Includes show-cards to assist with the interviews

### Arthritis status: self-reported and symptom-based

For the current analyses, self-reported diagnosis of arthritis (lifetime) was based on participant responses to the question; *“Have you ever been diagnosed with/told by a health care professional you have arthritis (a disease of the joints; or by other names rheumatism or osteoarthritis)?”* As a secondary endpoint, a symptom-based determination of arthritis (yes/no for current within the previous 12 months) was also employed, by applying an algorithm developed by the WHO SAGE study team [23]; questions and the algorithm are presented in Table 2.

**Table 2:** Symptom-based questions and the related algorithm to ascertain prevalent arthritis, developed as part of the World Health Organization SAGE Wave 1 [23]

Question number	Question text and algorithm
1	During the last 12 months, have you experienced pain, aching, stiffness or swelling in or around the joints (like arms, hands, legs or feet) which were not related to an injury and lasted for more than a month?
2	During the last 12 months, have your experienced stiffness in the joint in the morning after getting up from bed, or after a long rest of the joint without movement?
3	Did this stiffness last for more than 30 min?
4	Did this stiffness go away after exercise or movement in the joint?
Algorithm	If a participant responded with ‘yes’ to questions 1 and/or 2, and responded with ‘yes’ to question 3 and ‘no’ to question 4, then the participant was categorised as having arthritis

### Socioeconomic position

SEP was measured using two key parameters of marital status and educational attainment: the latter used due to the inextricable link between education and skilled vs. unskilled labour, and thus financial remuneration for work. Self-reported marital status was categorised for analyses into three groups of: (i) never married, (ii) currently married or cohabitating, and (iii) separated/divorced or widowed. Participants were asked if they had ever been to school; for those that indicated ‘yes’, they were also asked to identify the highest level of education completed. Education was categorised as (i) ‘no formal schooling’, (ii) less than primary school, or primary school completed, (iii) secondary school completed, or high school (or equivalent) completed, or (iv) college, pre-university or university completed, or post-graduate degree completed. Education levels were mapped to an international standard [24].

### Statistical analyses

Arthritis (self-reported and symptom-based) prevalence and 95% confidence intervals (95%CI) were calculated by implementing household level analysis weights separately for each of the six countries across 10-year age strata (the 20–29 year age group was expanded to also include those aged 18–19 years), sex, marital status and education. Country-specific survey weightings were applied, and weighted prevalence calculated for each country. Adjustment of prevalence estimates for differences in the age structure across countries was accomplished by age-standardisation, using the direct method of standardisation [25] and the WHO World Standard Population distribution (%) as standard population [26]. Ten-year intervals were used for age categorisation.

## Results

Country-specific numbers and proportions of the total 44,747 participants (total 57.1% women), were; China *n* = 15,050 (33.6%), Ghana *n* = 5573 (12.5%), India *n* = 12,198 (27.3%), Mexico *n* = 2752 (6.1%), the Russian Federation *n* = 4947 (11.1%), and South Africa *n* = 4227 (9.5%). Across the entire study population, 5003 women and 2664 men had (lifetime) self-reported arthritis (19.9% and 14.1%, respectively), whilst 1220 women and 594 men were identified as having (within previous 12 months) symptom-based arthritis (4.8% and 3.1%, respectively).

Table 3 presents the country-specific proportional responses (non-weighted) to the four symptom-based questions (see Table 2), that were included in the algorithm to determine symptom-based arthritis. For women, proportions that reported ‘any pain during the last 12 months’ or ‘any stiffness during the last 12 months’ were lowest for Mexico (28.4% [95% CI 26.3%–30.9%] and 23.3% [95% CI 20.9%–26.0%], respectively) and highest for the Russian Federation (48.4% [95% CI 46.4%–50.4%] and 50.5% [95% CI 48.8%–52.1%], respectively). For men, the proportions that reported ‘any pain during the last 12 months’ or ‘any stiffness during the last 12 months’ were lowest for Mexico (20.1% [95% CI 17.5%–23.0%] and 16.1% [95% CI%CI 14.1%–18.3%], respectively) and highest for the Russian Federation (32.9% [95% CI 30.5%–35.5%] and 34.6% [95% CI 32.4%–36.9%], respectively).

**Table 3:** Responses to the four questions^a^ included in the algorithm for symptom-based arthritis, stratified by country and sex^b^ (non-weighted)

Women (*n* = 25,180)						
	China (*n* = 8016)	Ghana (*n* = 2749)	India (*n* = 7489)	Mexico^b^ (*n* = 1692)	Russian Federation (*n* = 2806)	South Africa (*n* = 2428)
^c^Any pain during last 12 months? (Yes)	29.1% (28.0%–30.2%)	38.2% (36.4%–40.0%)	29.2% (28.0%–30.4%)	28.4% (26.3%–30.9%)	48.4% (46.4%–50.4%)	36.5% (34.6%–38.4%)
^c^Any stiffness during last 12 months? (Yes)	24.2% (23.2%–25.2%)	43.5% (41.5%–45.6%)	29.7% (28.5%–30.8%)	23.3% (20.9%–26.0%)	50.5% (48.8%–52.1%)	33.2% (31.2%–35.3%)
^d^Did stiffness last for >30 min? (Yes)	24.7% (22.4%–27.1%)	38.1% (35.6%–40.7%)	33.3% (30.9%–35.2%)	26.1% (21.8%–31.0%)	45.3% (42.8%–47.9%)	36.3% (33.3%–39.4%)
^d^Did stiffness go away after movement? (No)	19.2% (17.4%–21.0%)	31.5% (28.9%–34.2%)	25.4% (23.7%–27.3%)	15.3% (12.3%–18.9%)	33.1% (30.5%–35.9%)	19.8% (17.2%–22.7%)
Men (*n* = 18,914)						
	China (*n* = 6993)	Ghana (*n* = 2816)	India (*n* = 4709)	Mexico (*n* = 1050)	Russian Federation^b^ (*n* = 1549)	South Africa (*n* = 1797)
^c^Any pain during last 12 months? (Yes)	20.4% (19.6%–21.3%)	25.2% (23.5%–26.9%)	23.4% (22.0%–24.7%)	20.1% (17.5%–23.0%)	32.9% (30.5%–35.5%)	25.3% (23.3%–27.5%)
^c^Any stiffness during last 12 months? (Yes)	17.2% (16.4%–17.9%)	29.8% (28.2%–31.5%)	25.4% (24.1%–26.7%)	16.1% (14.1%–18.3%)	34.6% (32.4%–36.9%)	23.7% (21.9%–25.5%)
^d^Did stiffness last for >30 min? (Yes)	26.5% (24.4%–28.8%)	29.2% (25.6%–33.1%)	29.0% (26.5%–31.6%)	25.9% (19.7%–33.3%)	40.0% (35.0%–45.1%)	30.1% (25.6%–35.0%)
^d^Did stiffness go away after movement? (No)	20.4% (18.1%–22.9%)	25.2% (22.3%–28.3%)	22.5% (19.8%–25.4%)	17.9% (12.6%–24.8%)	29.4% (25.5%–33.7%)	16.4% (13.0%–20.6%)

Data presented as proportions with 95% confidence intervals (95% CI)

^a^Complete wording of the symptom-based questions are presented in Table 2

^b^Approximately 12% of the sample from the Russian Federation had no information regarding sex of respondents

^c^Proportions (95% confidence intervals) are based on the total study population from each LMIC

^d^Proportions (95% confidence intervals) are based on those that responded ‘yes’ to either one or both of the first two symptom-based questions

Table 4 presents the country-specific and sex-stratified prevalence of self-reported arthritis (weighted), across age strata, educational attainment and marital status. For both sexes in each country, arthritis prevalence increased proportionally with advancing age; with the exception of women from China and men and women from South Africa who had the greatest prevalence in the age group of 60–69 years, all other groups showed a peak in arthritis prevalence in the oldest age group ≥70 years. For women, the prevalence by country ranged from 22.9% (95% CI 11.2%–41.1%) in Mexico to 45.7% (95% CI 39.1%–52.3%) in the Russian Federation. For men, prevalence ranged from 9.7% (95% CI 6.3%–14.5%) in Mexico to 37.8% (95% CI 30.3%–46.0%) in the Russian Federation. In each country, women who had never been formally schooled or had completed less than primary school had the highest prevalence of arthritis compared to those with a greater level of educational attainment. Higher arthritis prevalence was consistently observed for women that were separated, divorced or widowed (range: Russian Federation 36.4% [95% CI 29.1%–44.4%] to Ghana 11.7% [95% CI 8.9%–15.1%]) compared to those that were never married or currently married (range: China 0.9% [95% CI 0.3%–3.0%] to South Africa 12.1% [95% CI 5.5%–24.7%]). Similar to women, men that had never been formally schooled had the highest arthritis prevalence, with the exception of men from the Russian Federation, for whom the greatest prevalence was observed in those that had completed all or some primary school level education (39.6% [95% CI 21.3%–61.4%]), however these numbers were small. Compared to other categories, men that were never married had the lowest arthritis prevalence (range: Mexico 0.1% [95% CI 0.0%–0.5%] to India 3.9% [95% CI 1.5%–9.5%]). In China and India, men that were currently married had the highest prevalence (11.9% [95% CI 9.4%–14.8%], and 8.8% [95% CI 7.2%–10.7%], respectively), whilst for all other countries, men that were separated, divorced or widowed were observed to have the highest arthritis prevalence (highest: Russian Federation 33.5% [95% CI 13.3%–62.3%]).

**Table 4:** Country-specific self-reported arthritis prevalence (weighted), across age strata, educational attainment and marital status, stratified by sex

	Women with self-reported arthritis (*n* = 5003)					
	China *n* = 1851	Ghana *n* = 350	India *n* = 946	Mexico *n* = 206	Russian Federation *n* = 1049	South Africa *n* = 601
Age (years)						
18–29	3.7% (0.9%–14.5%)	4.4% (1.3%–13.8%)	2.9% (1.9%–4.2%)	0.4% (0.1%–2.8%)	4.0% (0.6%–22.1%)	8.9% (1.8%–34.2%)
30–39	6.0% (3.8%–9.5%)	3.0% (0.9%–9.2%)	8.5% (6.7%–10.7%)	1.8% (0.5%–6.0%)	14.7% (7.0%–28.3%)	0.2% (0.0%–1.6%)
40–49	15.1% (11.2%–20.0%)	3.6% (1.6%–8.1%)	12.2% (9.6%–15.3%)	7.9% (2.2%–24.5%)	21.4% (10.5%–38.6%)	11.3% (5.6%–21.4%)
50–59	22.1% (20.0%–24.4%)	11.5% (9.1%–14.5%)	19.8% (16.7%–23.2%)	6.6% (2.3%–17.5%)	21.1% (15.6%–27.9%)	29.2% (24.6%–34.2%)
60–69	29.7% (27.1%–32.6%)	15.4% (12.1%–19.5%)	21.4% (16.7%–26.9%)	13.0% (8.8%–18.7%)	36.4% (29.6%–43.8%)	31.5% (25.7%–38.0%)
70+	29.2% (26.7%–31.9%)	22.8% (18.6%–27.6%)	23.5% (18.8%–29.0%)	22.9% (11.2%–41.1%)	45.7% (39.1%–52.3%)	26.5% (20.7%–33.2%)
Formal education^a^						
Never schooled	24.1% (19.9%–28.8%)	9.5% (7.0%–12.7%)	12.6% (10.9%–14.6%)	11.0% (4.7%–23.5%)	51.8% (31.0%–72.1%)	17.5% (12.8%–23.5%)
≤ Primary school	18.1% (13.7%–23.6%)	5.2% (2.9%–9.3%)	12.7% (10.5%–15.3%)	7.4% (3.7%–14.4%)	42.4% (33.0%–52.4%)	31.1% (21.0%–43.5%)
Secondary school	13.0% (10.1%–16.5%)	4.6% (2.4%–8.9%)	5.5% (4.0%–7.5%)	3.1% (1.3%–7.4%)	25.0% (20.0%–30.8%)	8.4% (4.8%–14.3%)
College	4.7% (1.6%–13.1%)	1.6% (0.7%–4.0%)	6.7% (2.7%–15.6%)	1.6% (0.7%–3.6%)	15.1% (10.0%–22.2%)	1.5% (0.6%–3.6%)
Marital status^b^						
Never married	0.9% (0.3%–3.0%)	7.8% (2.3%–23.2%)	1.1% (0.4%–3.0%)	1.3% (0.7%–2.4%)	7.8% (4.4%–13.4%)	12.1% (5.5%–24.7%)
Married	14.7% (12.6%–17.2%)	3.5% (2.1%–6.0%)	10.3% (9.1%–11.7%)	4.3% (2.5%–7.3%)	17.4% (12.4%–24.0%)	9.2% (5.5%–14.9%)
Divorced/widowed	25.2% (19.9%–31.5%)	11.7% (8.9%–15.1%)	19.1% (15.9%–22.7%)	19.0% (8.1%–38.4%)	36.4% (29.1%–44.4%)	19.3% (12.8%–28.1%)
	Men with self-reported arthritis (*n* = 2664)					
	China *n* = 1145	Ghana *n* = 230	India *n* = 578	Mexico *n* = 77	Russian Federation *n* = 363	South Africa *n* = 271
*Age strata (years)*						
18–29	1.3% (0.2%–8.8%)	−	2.1% (1.0%–4.7%)	−	−	0.7% (0.1%–3.4%)
30–39	5.5% (2.4%–12.1%)	0.2% (0.0%–1.4%)	6.1% (3.8%–9.8%)	−	14.6% (5.4%–34.1%)	1.3% (0.3%–5.8%)
40–49	12.0% (7.9%–18.0%)	3.7% (1.5%–8.7%)	7.9% (5.1%–12.1%)	2.9% (0.6%–13.2%)	4.7% (1.3%–15.9%)	0.9% (0.3%–3.0%)
50–59	13.7% (11.8%–15.8%)	7.4% (5.4%–10.1%)	13.7% (11.3%–16.5%)	0.9% (0.3%–2.6%)	21.6% (9.5%–42.2%)	12.6% (9.3%–16.8%)
60–69	20.0% (17.7%–22.5%)	11.6% (8.6%–15.4%)	16.9% (13.8%–20.6%)	8.0% (4.7%–13.3%)	21.3% (15.2%–29.0%)	28.2% (22.1%–35.2%)
70+	22.9% (20.7%–25.2%)	16.7% (12.6%–21.7%)	17.8% (14.5%–21.7%)	9.7% (6.3%–14.5%)	37.85 (30.3%–46.0%)	20.9% (13.5%–30.9%)
Formal education^a^						
Never schooled	22.3% (13.2%–35.2%)	6.3% (4.5%–8.7%)	9.0% (6.7%–12.1%)	7.7% (3.2%–17.3%)	4.4% (0.6%–26.8%)	10.4% (6.0%–17.6%)
≤ Primary school	14.8% (10.3%–20.7%)	2.9% (1.6%–4.9%)	9.0% (6.6%–12.1%)	3.7% (1.8%–7.7%)	39.6% (21.3%–61.4%)	7.1% (4.4%–11.2%)
Secondary school	9.2% (7.3%–11.5%)	4.6% (2.3%–8.9%)	8.5% (6.4%–11.3%)	0.3% (0.1%–0.7%)	11.9% (7.2%–19.0%)	2.2% (1.1%–4.6%)
College	7.4% (3.8%-13.95)	2.3% (1.1%–4.9%)	4.1% (2.0%–8.0%)	0.2% (0.0%–1.1%)	9.4% (3.1%–25.1%)	2.3% (0.9%–6.1%)
Marital status^b^						
Never married	3.0% (1.5%–5.9%)	0.3% (0.1%–1.0%)	3.9% (1.5%–9.5%)	0.1% (0.0%–0.5%)	0.9% (0.3%–3.1%)	2.0% (0.8%–4.7%)
Married	11.9% (9.4%–14.8%)	4.5% (3.1%–6.5%)	8.8% (7.2%–10.7%)	2.2% (1.1%–4.4%)	11.3% (7.5%–16.7%)	5.6% (4.1%–7.6%)
Divorced/widowed	11.8% (8.7%–15.7%)	8.5% (5.4%–13.2%)	6.5% (3.8%–10.7%)	6.6% (3.3%–12.6%)	33.5% (13.3%–62.3%)	13.2% (5.6%–27.9%)

Data presented as proportions with 95% confidence intervals (95% CI)

*Abbreviations*: *LMIC* low and middle income countries, *WHO* World Health Organization

^a^Categories of formal education are; ≤primary school (less than primary school, or primary school completed); secondary school (secondary school completed, or high school or its equivalent completed); college (college or pre-university completed, or post-graduate degree completed)

^b^Categories of marital status are; married (currently married or cohabiting); divorced/widowed (separated or divorced, or widowed)

Table 5 presents the country-specific and sex-stratified prevalence of symptom-based arthritis prevalence (weighted), across age strata, educational attainment and marital status, for each LMIC. Patterns of symptom-based arthritis prevalence were similar to self-reported arthritis for both sexes; however, prevalence was lower than observed for self-reported arthritis.

**Table 5:** Country-specific symptom-related arthritis prevalence (weighted) across age strata, educational attainment and marital status, stratified by sex

Women	With symptom-related arthritis (*n* = 1220)					
	China *n* = 201	Ghana *n* = 290	India *n* = 238	Mexico *n* = 29	Russian Federation *n* = 358	South Africa *n* = 104
Age (years)						
18–29	−	−	0.9% (0.4%–1.8%)	−	−	−
30–39	−	1.6% (0.4%–6.6%)	1.5% (0.7%–3.2%)	−	12.5% (4.4%–30.7%)	0.2% (0.0%–1.7%)
40–49	0.3% (0.1%–1.3%)	3.3% (1.3%–7.9%)	2.8% (1.7%–4.4%)	1.2% (0.2%–8.0%)	2.3% (0.5%–9.5%)	2.4% (0.3%–15.7%)
50–59	4.1% (3.0%–5.7%)	11.5% (8.6%–15.2%)	5.9% (43%–8.0%)	0.7% (0.1%–4.1%)	4.3% (2.6%–7.1%)	6.2% (3.7%–10.2%)
60–69	4.0% (2.8%–5.8%)	16.5% (12.3%–21.9%)	5.6% (4.0%–7.9%)	1.5% (0.7%–3.2%)	10.0% (7.0%–14.2%)	5.5% (3.0%–9.8%)
70+	5.6% (3.9%–7.9%)	18.6% (14.9%–23.0%)	6.7% (4.7%–9.7%)	2.1% (0.9%–4.7%)	20.1% (14.4%–27.4%)	5.6% (3.3%–9.2%)
Formal education^a^						
Never schooled	4.1% (3.3%–5.1%)	9.4% (6.9%–12.7%)	3.7% (2.9%–4.7%)	1.5% (0.5%–4.1%)	41.8% (16.6%–72.2%)	3.5% (1.7%–6.9%)
≤ Primary school	2.0% (1.3%–3.1%)	2.2% (1.4%–3.6%)	2.2% (1.5%–3.4%)	1.0% (0.3%–3.3%)	22.6% (14.3%–33.7%)	5.9% (2.3%–14.3%)
Secondary school	0.5% (0.3%–0.8%)	3.0% (1.3%–6.5%)	1.2% (0.6%–2.5%)	0.0% (0.0%–0.3%)	8.9% (4.9%–15.4%)	1.0% (0.4%–2.6%)
College	0.0% (0.0%–0.2%)	−	1.1% (0.2%–6.4%)	0.0% (0.0%–0.3%)	4.3% (2.4%–7.5%)	0.3% (0.1%–1.2%)
Marital status^b^						
Never married	−	1.9% (0.4%–9.0%)	1.1% (0.3%–3.7%)	0.3% (0.1%–0.9%)	1.7% (0.7%–4.2%)	2.6% (0.6%–11.2%)
Married	1.1% (0.9%–1.4%)	2.6% (1.7%–4.1%)	2.5% (1.9%–3.2%)	0.7% (0.2%–2.4%)	3.1% (1.9%–4.9%)	1.3% (0.6%–2.9%)
Divorced/widowed	4.2% (2.2%–7.9%)	10.8% (7.7%–15.0%)	4.8% (3.5%–6.7%)	0.6% (0.3%–1.4%)	18.0% (9.9%–30.5%)	3.4% (2.0%–5.6%)
Men	With symptom-based arthritis (*n* = 594)					
	China *n* = 138	Ghana *n* = 170	India *n* = 113	Mexico *n* = 15	Russian Federation *n* = 117	South Africa *n* = 41
Age strata (years)						
18–29	−	1.0% (0.1%–7.2%)	0.8% (0.1%–4.5%)	−	2.3% (0.3%–16.2%)	−
30–39	−	1.7% (0.5%–5.4%)	0.8% (0.2%–3.8%)	−	5.2% (1.0%–22.2%)	−
40–49	0.8% (0.2%–2.8%)	0.6% (0.1%–2.5%)	1.9% (0.8%–43%)	−	1.9% (0.2%–13.4%)	1.7% (0.4%–6.6%)
50–59	2.3% (1.6%–3.1%)	3.8% (2.7%–5.4%)	2.6% (1.1%–6.2%)	−	1.9% (0.9%–4.1%)	2.3% (1.0%–4.9%)
60–69	3.8% (3.3%–4.4%)	9.1% (6.7%–12.2%)	3.5% (2.0%–6.1%)	0.6% (0.2%–2.2%)	6.4% (3.3%–12.0%)	3.7% (1.8%–7.5%)
70+	4.3% (3.5%–5.2%)	9.2% (6.9%–12.3%)	4.8% (3.0%–7.5%)	3.0% (1.5%–5.9%)	10.7% (6.9%–16.4%)	6.0% (2.4%–14.3%)
Formal education^a^						
None	4.8% (3.3%–6.8%)	5.6% (4.0%–7.7%)	2.7% (1.3%–5.5%)	1.5% (0.5%–3.8%)	3.4% (0.3%–27.7%)	4.2% (1.5%–10.8%)
≤ Primary school	1.2% (1.0%–1.6%)	1.9% (1.1%–3.2%)	1.2% (0.7%–1.9%)	0.2% (0.1%–0.6%)	10.4% (5.0%–20.4%)	1.6% (0.8%–3.4%)
Secondary school	1.0% (0.4%–2.3%)	1.4% (0.6%–3.1%)	2.0% (1.0%–4.2%)	0.1% (0.0%–0.5%)	3.9% (1.7%–9.0%)	0.9% (0.2%–5.4%)
College	0.1% (0.0%–0.4%)	0.7% (0.2%–2.2%)	0.4% (0.1%–1.6%)	0.2% (0.0%–1.4%)	1.4% (0.4%–5.3%)	−
Marital status^b^						
Never married	0.9% (0.2%–5.0%)	1.5% (0.2%–8.8%)	1.6% (0.3%–8.2%)	−	0.2% (0.0%–0.9%)	0.8% (0.2%–2.8%)
Married	1.1% (0.6%–1.8%)	2.5% (1.8%–3.4%)	1.8% (1.1%–2.7%)	0.3% (0.1%–0.5%)	3.7% (1.7%–8.0%)	1.4% (0.6%–3.2%)
Divorced/widowed	2.9% (1.7%–4.9%)	6.5% (3.8%–10.9%)	1.9% (0.6%–5.5%)	0.6% (0.1%–2.6%)	7.9% (2.9%–19.6%)	2.9% (0.7%–11.4%)

Data presented as proportions with 95% confidence intervals (95% CI)

*Abbreviations*: *LMIC* low and middle income countries, *WHO* World Health Organization

^a^Categories of formal education are; ≤primary school (less than primary school, or primary school completed); secondary school (secondary school completed, or high school or its equivalent completed); college (college or pre-university completed, or post-graduate degree completed)

^b^Categories of marital status are; married (currently married or cohabiting); divorced/widowed (separated or divorced, or widowed)

**Fig. 1:**
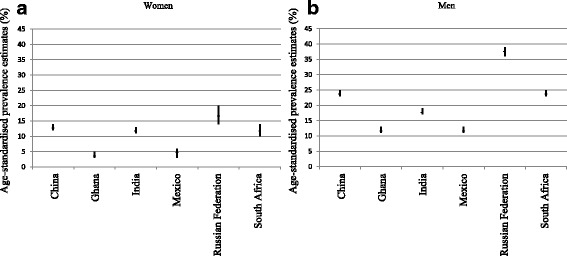
Box plot presenting the direct age-standardised prevalence estimates (%) and 95% confidence intervals of self-reported arthritis diagnosis for each of the lower to middle income countries, for women (**a**) and men (**b**)

Figure 1 presents a box plot of the age-standardised rates of self-reported arthritis, stratified by sex, across each country (crude and age-standardised rates are presented in Additional file 1: Online Table S1). For five of the six LMICs, the standardised rates of arthritis for men were approximately twice that observed for women; the exception was Ghana, where men had rates three times greater than those observed for women (12% [95% CI 11%–13%] vs. 4% [95% CI 3%–5%]). The highest rates of arthritis were observed in the Russian Federation: for men the rate was 38% (95% CI 36%–39%) and for women it was 17% (95% CI 14%–20%).

## Discussion

We present the prevalence of arthritis across age, sex and different parameters of SEP in a large population-based study spanning six LMICs. Across the countries and for both sexes, higher arthritis prevalence was consistently associated with older age and lower educational attainment, whilst higher prevalence was also observed in women, but not men, that were separated, divorced, or widowed.

The pattern between advancing age and increasing arthritis prevalence in LMICs appears similar to the pattern observed in higher income countries [27]. However, after age-standardisation, we observed in our current study that the rates of arthritis in LMICs were greater than those reported in higher income countries, specifically for men from China, India, the Russian Federation and South Africa. Compared to higher income countries, higher age-standardised rates of arthritis were also observed for women from the Russian Federation; however, for the remaining five LMICs, rates appeared to be similar to those observed from higher income countries. Our results indicate the importance of age-standardisation when reporting prevalence data, in order that fair comparisons can be applied when discussing whether any disparities in diseases exist between countries. In addition to the peak of arthritis prevalence observed in older age groups, we observed a sizeable proportion of arthritis in younger age groups; prevalence that would have significant impacts on work capacity and social roles. Indeed, whilst contextually different and focused upon osteoarthritis, similar findings have been reported in higher income countries [28, 29].

Ours are the first prevalence figures of arthritis to be presented across different socioeconomic parameters for residents of LMICs. Whilst the overall arthritis prevalence has been reported for some countries included the SAGE, specifically India [30] and China [31], we now present age-standardised prevalence across all six countries (Additional file 1: Table S1). Higher prevalence of arthritis among individuals with lower educational attainment in LMICs, may be indicative of the inextricable link between lower education and lower-skilled, highly manual labour. Furthermore, these findings also replicate the association observed in higher income countries. For instance, lower educational attainment has been associated with the prevalence of many chronic diseases, including self-reported arthritis (non-specific) [32], osteoarthritis [33] and rheumatoid arthritis [34]. Our observation of higher prevalence of arthritis in individuals that were divorced, widowed or separated, may be related to those persons also more likely to be older. However, and whilst speculative, it may plausibly be due to having a greater workload that cannot shared with a partner. Should these individuals also have lower educational attainment, any job-related exposures will likely be manual and thus with greater biomechanical stress on the joints due to increased exposure to heavy lifting, repetitive movements and/or squatting [35, 36].

The prevalence of musculoskeletal diseases per se in LMICs [3] will potentially have a greater impact than in high income countries due to the reduced capacity of LMICs to avoid and/or alleviate the impact at individual and national levels. This is especially pertinent given that global NCD initiatives do not list musculoskeletal diseases within the ‘top four’ [3]. In LMICs where pain management is less than optimal [37], the burden of chronic, and possibly untreated, pain will be compounded by social and environmental stressors that require individuals work and fulfil community roles regardless of pain. Indeed, data from a WHO collaboration reported that between 5 and 33% of individuals in LMICs experience chronic pain on a daily basis [38]. Similarly, we observed a sizeable proportion of respondents to have stiffness lasting longer than 30 min and which did not alleviate with movement; these characteristics are indicative of chronic pain, and potentially suggest inflammatory arthropathy. In addition, diseases such as fibromyalgia are likely to cause joint pain, however, we are unable to determine if this, and similar issues, may have biased responses to symptomatology-related questions. Any ‘treatment gap’ is at odds with the WHO Constitution, which recognises “…the highest attainable standard of health as a fundamental right of every human being” [39], however, LMICs experience a disproportionately lower likelihood of achieving that standard. We speculate that resource-poor populations, where ‘informal workers’ are central to community structure, are most at risk of worsening poverty levels due to increased YLD attributable to highly prevalent, and potentially undertreated, arthritis. It is important to note that whilst the burden of non-communicable diseases is increasing, there is a concurrent decline in the burden of infectious diseases [2]. Given this, more attention must be given to the management of diseases such as arthritis in LMICs: action on musculoskeletal diseases per se in LMICs present opportunities for such action [3]. Models of care (MoC) for musculoskeletal diseases have been developed and implemented in the LMICs of The Philippines, Malaysia, Bangladesh and Myanmar [40]. Despite mixed results, a four-step process was designed to inform future development of musculoskeletal-related MoC for implementation in LMICs; (i) identify the scale of the problem, (ii) identify the need, (iii) develop the action plan (including community engagement and addressing workforce capacity), and (iv) employ a coordinated approach to implementing the intervention program/MoC [40].

Despite advances in diagnosis and treatment of arthritis during the last few decades in higher income countries [16], these advances have not impacted on LMICs, which are primarily resource-poor. Gross domestic product and health care expenditures per capita are strongly correlated [14, 41]. Governments in LMICs are constrained by competitive social, economic, health- and poverty-related issues [7]; this frequently results in chronic diseases such as arthritis achieving lower priority when urgent health needs are considered in an environment with poor education, scarce resources, and rapid population growth [7, 42]. Not only is suboptimal access to healthcare a concern, but the cost of healthcare may be many-fold the gross domestic product, and thus unattainable for the majority of the population of LMICs [5]. For many individuals and households in LMIC, there are inadequate financial resources to manage the cost of chronic disease, with an impoverishing effect of paying for healthcare services out-of-pocket [43]. In order to address the problem of out-of-pocket healthcare expenses, the WHO is encouraging countries to provide universal health coverage [7]. For LMICs the provision of universal health coverage may be in the form of community-based health insurance schemes, whereby the community voluntarily raises, pools, allocates, purchases and supervises the health financing arrangement [7, 44]. Whilst there are some national efforts to prioritise healthcare resources and achieve universal health coverage, these schemes are likely to focus on supporting healthcare for diseases that cause early mortality rather than those that result in disability.

Our study has a number of strengths. The SAGE study consists of a large multi-national cohort, and our population for this analysis encompassed almost 45,000 participants. The integrity and coordination of these data is overseen by WHO, in close collaboration with leading research institutions in each of the countries, and with a level of involvement from national health authorities [23]. The use of a standardized survey instrument and methods for SAGE Wave 1, the recruitment of representative samples, and the application of country-specific weightings to calculate our prevalence estimates have enabled comparison with similar surveys conducted in higher income countries. In addition, the use of standardized tools to measure SEP in each of the countries in SAGE enables us to undertake between-country comparisons. Our findings build on the prevalence data reported by the GBD Study, whereby estimates were based on systematic reviews of published data on incidence, prevalence, and severity; however, for some LMICs only limited data were available [45]. Our study also builds on previous analyses using the SAGE dataset, as no study to date has presented arthritis prevalence figures across parameters of SEP.

This study also has some limitations. We acknowledge that SAGE chronic disease data are self-reported, and thus may be subject to recall bias and potential inaccuracy with a subsequent uncertainty of estimates. However, the self-reported arthritis question is similar to that used for other large population level studies, including those reported by the Centers for Disease Control Arthritis Program in the United States [46], and self-reported arthritis has also been reported as a sensitive measure for public health surveillance [47]. It is possible that limited access to healthcare professionals in LMICs may lead to an underestimation of arthritis prevalence, and those who have arthritis but have not yet sought care may have been missed. In addition, it may be possible that in many countries diagnoses of arthritis may be made by a non-medical healthcare provider, thus introducing some ambiguity in responses to the diagnosis question. Yet here, the symptom-reported prevalence, where access to healthcare professionals would be removed from the equation, indicated an even lower burden of arthritis than by self-reported diagnosis; an issue that may also be related to diagnosed arthritis being across the lifetime, whilst symptom-based arthritis was within the previous 12 months. Our study does not link prevalence data with disability; however it should be noted that arthritis has highly variable impacts on the person. A high proportion of SAGE Wave 1 participants indicated that they had no formal education (~50%); this may explain the level of missing data pertaining to the ‘highest level of educational attainment’ variable. However, missing data may also be attributable to the WHO data collection ‘Individual Questionnaire’ tool, which did not include a category for those that had completed primary school but who had not completed secondary school. It has been reported that, in several countries, urban dwellers were more likely to refuse to participate in SAGE [23], which may present a bias toward rural-based participants; however, the high proportion of rural residents may conversely be considered a key strength of the SAGE dataset as non-metropolitan groups are commonly under-represented in population-based surveys. We acknowledge that the six countries differ substantially in terms of culture, society, and healthcare system, and thus our pooled estimates should be considered in this light. Finally, the response rates were relatively low for Mexico (53%), due to a short time-frame for data collection, although response rates for all other countries in SAGE Wave 1 were 75% or greater, with the exception of India at 68% [23].

## Conclusions

In conclusion, we have identified a high prevalence of arthritis in LMICs. For people living in LMIC, functional ability and mobility is imperative to survival, and our findings therefore have implications for prioritising healthcare resources toward arthritis prevention and treatment in relatively resource-poor countries. It is plausible that, especially for residents of LMICs, the high prevalence of arthritis may limit their ability to financially and/or materially support themselves. Similarly, poverty and lower educational attainment may predispose populations to manual labour, and subsequent predisposition to diseases such as osteoarthritis. Future work will focus on occupational types and occupational activities as risk factors for arthritis and related symptomatology. Our current findings have implications for national efforts to achieve universal health coverage and to prioritise healthcare resources toward preventing and/or treating arthritis.

## Additional file

Additional file 1: Online Table S1. Crude and direct age-standardised prevalence estimates (95%CI) of arthritis, stratified by sex. (DOCX 12 kb)

## Abbreviations

GBD: Global burden of disease study
LMICs: Lower and middle income countries
MoC: Models of care
NCD: Non-communicable disease
SAGE: Study on global AGEing and adult health
SEP: Socioeconomic position
WHO: World Health Organization
YLD: Years lived with disability
