# Supplementary material for: Prevalence of arthritis according to age, sex and socioeconomic status in six low and middle income countries: analysis of data from the World Health Organization study on global AGEing and adult health (SAGE) Wave 1
Source: BMC Musculoskelet Disord. 2017 Jun 21;18:271. doi: 10.1186/s12891-017-1624-z (PMC5479046; doi:10.1186/s12891-017-1624-z)
Supplement: Additional file 1: — Online Table S1. Crude and direct age-standardised prevalence estimates (95%CI) of arthritis, stratified by sex. (DOCX 12 kb) [file 12891_2017_1624_MOESM1_ESM.docx]

**Online Supplementary Table 1:** Crude and direct age-standardised prevalence estimates (95%CI) of arthritis, stratified by sex

|  | **Women** | | | **Men** | | |
| --- | --- | --- | --- | --- | --- | --- |
|  | n | Crude rate | Adjusted rate (95%CI) | n | Crude rate | Adjusted rate (95%CI) |
| *China* | 13,440 | 0.02 | 0.13 (0.12-0.14) | 15,120 | 0.25 | 0.24 (0.23-0.25) |
| *Ghana* | 3,640 | 0.06 | 0.04 (0.03-0.05) | 5,713 | 0.13 | 0.12 (0.11-0.13) |
| *India* | 9,318 | 0.13 | 0.12 (0.11-0.13) | 7,942 | 0.18 | 0.18 (0.17-0.19) |
| *Mexico* | 1,256 | 0.06 | 0.05 (0.03-0.06) | 3,754 | 0.13 | 0.12 (0.11-0.13) |
| *Russian Federation* | 3,290 | 0.22 | 0.17 (0.14-0.20) | 4,930 | 0.42 | 0.38 (0.36-0.39) |
| *South Africa* | 3,700 | 0.19 | 0.12 (0.10-0.14) | 4,290 | 0.24 | 0.24 (0.23-0.25) |

**Abbreviations:** 95%CI = 95% confidence intervals
